# Exploring Holocene Changes in Palynological Richness in Northern Europe – Did Postglacial Immigration Matter?

**DOI:** 10.1371/journal.pone.0051624

**Published:** 2012-12-11

**Authors:** Thomas Giesecke, Steffen Wolters, Susanne Jahns, Arthur Brande

**Affiliations:** 1 Department of Palynology and Climate Dynamics, Albrecht-von-Haller-Institute for Plant Sciences, Georg-August-University Göttingen, Göttingen, Germany; 2 Lower Saxony Institute for Historical Coastal Research, Wilhelmshaven, Germany; 3 Brandenburg Department of Cultural Heritage and Archaeological State Museum, Zossen, Germany; 4 Department of Ecology, Technical University Berlin, Berlin, Germany; New York State Museum, United States of America

## Abstract

In mid to high latitudes glacial and interglacial cycles have repeatedly changed the area available for plant growth. The speed at which plants are able to colonize areas at the onset of an interglacial is hypothesized to limit their distribution ranges even today (migrational lag). If the spread of plants would have been generally slow then plant diversity in previously glaciated areas would be expected to increase over time. We explore this hypothesis using results from six palynological investigations from two previously glaciated regions: central Sweden and north-eastern Germany. Rarefaction, slope of rank order abundance, and taxa accumulation plots were used to evaluate richness and evenness in pollen data in an attempt to separate richness from evenness. These analyses show little change in palynological richness for the northern sites throughout the Holocene. In contrast, the southern sites show an increase in richness and evenness during the early Holocene; this may be explained by the different initial conditions at the onset of the Holocene. A strong rise in palynological richness around 6000 and 1000 years ago at the southern sites can be attributed to the regional initiation of agriculture and major opening of the forest, respectively. For the northern sites there is no evidence for increased taxonomic diversity through time that could be due to delayed immigration of species.

## Introduction

To forecast the impact of climate change on biological diversity it is crucial to have knowledge on the ability of plants to shift their distribution in response to climate change [1]. Until recently, many studies suggested that plants will not be able to track climate change [2]. New research indicates that plants are moving fast in response to a warmer climate [3]. However, monitoring periods are too short to evaluate the effects of ecosystem resilience, species adaptation and rare long distance dispersal events [4], [5]. Therefore insights from the late Quaternary history of ecosystems are essential to help answer this question [6]–[8]. The biodiversity of the temperate forests are shaped by glacial-interglacial cycles that repeatedly changed the habitat available to plants [9]. Studying the patterns and processes of changing plant distributions at the end of glacial periods will also help to understand the spatial differences in biodiversity [10], [11].

The cold and dry climate of the glacial period expelled plants from many areas where they occur today. Continental ice sheets covered large parts of the northern hemisphere, eliminating any plant growth except possibly on isolated nunataks [12]. Thus nearly the complete flora that we find in previously glaciated areas today, must have spread into these regions sometime between deglaciation and the present day. It has long been assumed that the distances between Last Glacial Maximum (LGM) occurrences to present-day occurrences would affect the timing when the species in question appeared in a particular region [13], [14]. The time span - between the time that a taxon could have been present at a location due to climate warming and/or deglaciation and its first appearance - has been referred to as migrational lag.

The application of species distribution models to present-day climate suggested that many species did not fill their climatically described range [15], rejuvenating the question whether species limits are controlled by slow spread out of their LGM distributions [16], [17]. On the other hand, the rapid spread of alien species, like *Senecio inaequidens* in Europe [18], shows that herbs may expand their ranges over hundreds of kilometres in few decades.

Compilations of pollen and macrofossil investigations have been used to infer distributional changes of major tree species indicating that some trees assumed their current range late during the Holocene and may still be in the process of extending their distribution [14]. However, it is difficult to decide whether these boundary shifts were controlled by Holocene climate change [19]–[21], land-use change [22] or limited seed dispersal and time required to reach reproductive age [23], [24].

While compilations of pollen diagrams have revealed detailed accounts on the Late-Glacial and Holocene histories of mainly trees and shrubs [25], [26], little comparative use has been made of the many other pollen types that are recognised in pollen analytical investigations (but see [27]–[29] among others for regional comparisons). The reason for the lack of sub-continental comparisons lies in relating the number of distinct pollen types in the sample (palynological richness) to plant species richness in the area around the sample location. This problem has two major causes: differences in pollen production and dispersal among species and restricted taxonomic resolution in pollen analysis where identification often stops at the genus and sometimes even at the family or subfamily level [30]. Regardless, many pollen diagrams depict more than 100 pollen taxa and the pollen key for central Europe and adjacent areas by Beug [31] differentiates 586 types based on 2500 investigated species of flowering plants. Thus a substantial proportion of floristic richness is captured by palynological richness, even if this representation is biased by large families like Poaceae that yield a single pollen type for almost all non-cereal grasses.

The aim of this study is to explore the potential effect of delayed or slow plant spread on palynological richness through time. Different approaches to evaluate changes in palynological richness through time are considered and new analyses are suggested.

If migrational lag is an important mechanism that shaped current plant distributions, it should have worked gradually through time, increasing the floristic richness of a region. As a consequence palynological richness should have been low shortly after deglaciation. The increase in the number of species may slow or stop at some point in time, when all species with potential occurrence have reached the region. As a general process this should have occurred across all floristic geoelements and therefore be visible in the palynological record. As dispersal mechanisms differ between taxonomic groups an ordered arrival could be expected.

Additional factors potentially influencing diversity through time are changes in climate and human land-use, which are difficult to separate. However, the trend and magnitude of both factors are well documented for central and northern Europe so that their influence on changes in palynological richness can be evaluated.

Thus, by analysing and comparing palynological richness in selected pollen diagrams with good taxonomic resolution, we can explore whether migrational lag is an important mechanism that shaped floristic richness in previously glaciated areas. Finding a gradual increase in the number of pollen types through the Holocene, particularly in recently deglaciated areas would point towards an importance of migrational lag, unless it may be caused by the action of man or climate change. A lack of such an increase would indicate that the effect of migrational lag is negligible on a timescale of hundreds to thousands of years.

## Methods

### Site Selection and Additional Resources

**Figure 1:**
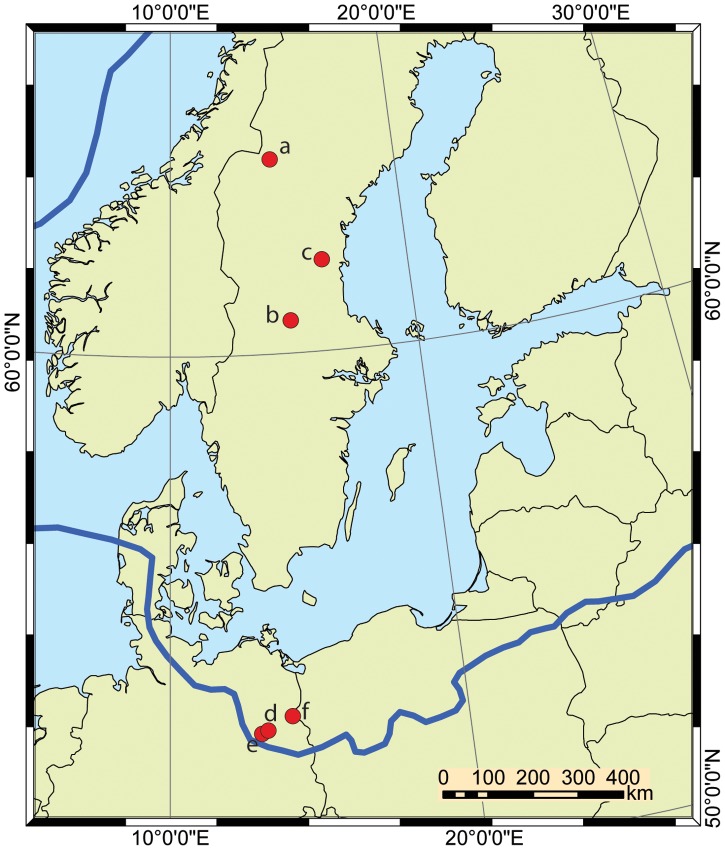
Location of pollen diagrams used in the analysis. Abborrtjärnen = a, Holtjärnen = b, Klotjärnen = c, Tegeler See = d, Schwanengraben = e, Krebssee = f. The blue line marks the approximate limit of ice cover at the Last Glacial Maximum [30].

Published pollen diagrams were selected from two regions with different climate that were glaciated during the last ice age [32] (Figure 1) and are home to similar forests in structure and species composition. As the degree of taxonomic differentiation in standard counts varies between investigators diagrams were selected from authors coming from the same palynological school to allow between-site comparisons. Pollen diagrams from three small lakes were selected from north-central Sweden, an area that was ice covered until the early Holocene. Holtjärnen and Klotjärnen [33] are today situated in the southern boreal forest. The area around Holtjärnen became ice free around 10600 years ago. After the retreat of the glacier, the vicinity of Klotjärnen was submerged and through isostatic uplift became a peninsula reaching into the Baltic Sea at about 9500 years ago. Abborrtjärnen is situated further north in the mid Boreal forest and the record starts around 9700 years ago, soon after deglaciation [34].

Northeast Germany was ice covered during the southernmost extent of the last glaciation and the area was ice free for about 5000 years before the beginning of the Holocene. Krebssee is of similar type and size as the Swedish lakes and situated one kilometre away from the river Oder [35]. Tegeler See is a large lake exceeding 1 km^2^, but the diagram [36] comes from a bay with a diameter of 200 m. Schwanengraben is an elongated depression about 50 m broad and several hundred meters long, which was a lake during the Late-Glacial and early Holocene and developed into a bog around 9000 years ago [37].

Pollen diagrams for these sites with a standardized selection of taxa are provided in Figure S1 (Supporting Information). The pollen counts for the six sites are available from the Open Access library Pangaea under the following Digital Object Identifier: Abborrtjärnen (doi:10.1594/PANGAEA.738429), Klotjärnen (doi:10.1594/PANGAEA.739382), Holtjärnen (doi:10.1594/PANGAEA.739308), Tegeler See (doi:10.1594/PANGAEA.792760), Schwanengraben (doi:10.1594/PANGAEA.792758), Krebssee (doi:10.1594/PANGAEA.792783, doi:10.1594/PANGAEA.792782, doi:10.1594/PANGAEA.792781).

Pollen counts are generally exceeding 1000 grains per sample for the northern sites and in most samples from Schwanengraben. For Tegeler See and Krebssee the counts range between 1000 and 2000 grains and between 600 and 1500, respectively. The raw pollen counts from the six sites were reduced to taxa exclusively coming from upland vascular plants to reduce site specific changes in the aquatic and telmatic environment (set A). A further restriction, excluding pollen types that come from archaeophytes or neophytes (see Table S1 in Supporting Information) aims to reduce the obvious impact that human land-use had on regional plant diversity (set B). Unless otherwise indicated the analysis were carried out on set A.

In addition to the selected three pollen diagrams from northeast Germany, further information on the first appearance of selected pollen taxa in this region was obtained from a local database. Pollen analytical investigations carried out between 1974 and 2000 at the department of Ecosystem Science and Plant Ecology at the Technical University Berlin were partly collected into a database. Investigations were initially restricted to the area of West-Berlin with the addition of sites in Brandenburg after 1990. The intention of the database was to collect information on the first occurrence of pollen types from herbaceous vegetation and selected trees. Pollen types of common trees and those of higher taxonomic level (genera, families) have not been included (Table S2 in Supporting Information). The database contains information from 113 sites with varying sample number. Samples were assigned to well-recognizable regional pollen zones [36], which have been radiocarbon dated at a number of sites [35].

### Extracting Diversity Information from Pollen Data

Pollen diagrams are mainly produced from sediments that accumulate in lakes and wetlands, and only where the focus of the investigation is this particular ecosystem, palynological richness can be directly used to describe for example the species diversity of water plants in a lake. Pollen that reaches the lake or wetland from beyond its limits does not have a defined area of origin. The probability of a pollen grain to be deposited at a site decreases with distance of the parent plant from the site. In absolute terms this differs largely between plants depending on pollen production and dispersal properties. Thus palynological richness cannot be related to a particular area and may be best compared to the regional species pool (gamma diversity). It depends heavily on the size of the pollen count [38], but there is no natural threshold that would indicate how many pollen grains should be counted per sample. In combination with the taxa specific pollen productivity and dispersal characteristics, this means that diversity measures including abundance are biased and potentially erroneous [39]. This leaves palynological richness itself as an important diversity measure for pollen data, which has to be expressed to a standard number of pollen grains counted to make it comparable between samples. Where pollen sums differ between samples or sites this is achieved using the rarefaction technique [29]. However, the evenness of a pollen sample determines the number of pollen types that may be encountered at a given pollen sum [30], [40]. Peros and Gajewski [41] find a positive correlation between palynological richness and evenness and a negative trend for palynological richness and pollen concentration in a surface sample dataset from the Canadian arctic. Changes in pollen concentration can be caused by changes in sedimentation rate and thus pollen accumulation rates should be used in such comparisons [42]. Using pollen accumulation rates, it is theoretically possible to overcome the effect of differential pollen productivity [42]. However, such estimates are highly dependent on the accumulation rate of the sediment which in turn can usually only be estimated with a high uncertainty. Thus in most cases the uncertainty will be larger than the signal, unless the focus of the investigation is the change in diversity from vegetation with high pollen production to one with low pollen productivity.

### Diversity and Evenness Indices and Analyses

The richness of pollen types and their equitability in abundance are strongly connected. Depending on the size of the pollen sum, palynological richness is more influenced by one or the other [43]. Using rarefaction, palynological richness was calculated to a basis of 500 pollen grains from vascular upland plants, which is assumed to reveal in particular the richness of pollen types in the sample.

As with estimates of richness, indices of evenness from samples with different count size are potentially biased. For this reason we used two indices that are not affected by differences in sample size. Pollen sample evenness was calculated using a modified version of the E_Q_ index [44]. A sample based threshold of 0.3% was applied for the inclusion of taxa to avoid the influence of single finds, which makes it independent of the number of grains counted. For the remaining taxa the slope of the regression between the proportion of rank order and the logarithm of proportional abundance (b’) was transformed according to Smith and Wilson [44]:

E_Q_ = –2π^−1^ × arctan(b′).

Rarefaction to low pollen sums (10–50) may also be a good indicator for pollen sample evenness, as abundant types will dominate such a small sample and the chance to encounter less abundant types is low [43]. Here we use rarefaction to a count of 30 grains as a comparison to the above evenness index.

Finding a pollen type in one sample but not in the neighbouring samples does not necessarily mean that the parent plant only occurred in the surroundings of the site during that particular time. For pollen types from plants with low productivity it may be safe to assume that the plant also occurred in the area before and after the period for which the pollen type was encountered. Little use has been made of this concept in Quaternary palynology, while it is readily applied to studies of older sediments [45]. To capture the information carried by sporadically occurring pollen types, counts were combined over consecutive samples into periods of 2000 years and 1500 years for the youngest period. All Late-Glacial samples were combined for each of the southern sites. These combined samples were used to calculate the number of taxa common or changing between periods, yielding estimates of beta diversity through time. In these combined samples, the number of taxa encountered still depends on the overall number of pollen counted and rarefaction was calculated to the lowest combined count for the respective site. The analysis was subsequently carried out for a combination of the northern and southern sites, respectively, omitting pollen types from archaeophytes and neophytes (set B) and using time periods of 1000 years.

In pollen counts, as in floristic surveys the number of pollen types and species increases with the pollen sum and sampling effort, respectively. However, in floristic surveys with defined area it is theoretically possible to find all species. Thus the relationship between the accumulated number of species encountered and sampling effort should follow an asymptote and the total number of species may be estimated [46]. Weng et al. [47] suggested that such an asymptote may exist in palynological data as well but point out that it has not yet been observed.

Here we calculated the accumulation of taxa over consecutive samples, which represents a species time relationship [48], while it also captures the increased sampling effort, as the number of pollen counted was also accumulated and varies between samples. Assuming taxa were not lost over the course of the Holocene, the curve can give insights on a changing species pool through time and this relationship may help to separate pollen type richness from evenness. Samples were accumulated in chronological order, beginning with the oldest sample from the northern sites and the first Holocene sample in the southern sites. The linear relationships in logarithmic space were described by linear regression models using the same slope but different intercept parameters. The residuals between the model and the observations were calculated in normal space and plotted against time.

A weak lowess smoother was applied to all results yielding scattered values to improve visual comparison without removing too much variance. Lowess was applied with a span of 0.2 to the rarefaction and evenness analysis and a span of 0.1 to the residuals from the taxa accumulation models. All calculations and computations were carried out using the R platform [49] and the vegan package [50].

## Results

**Figure 2:**
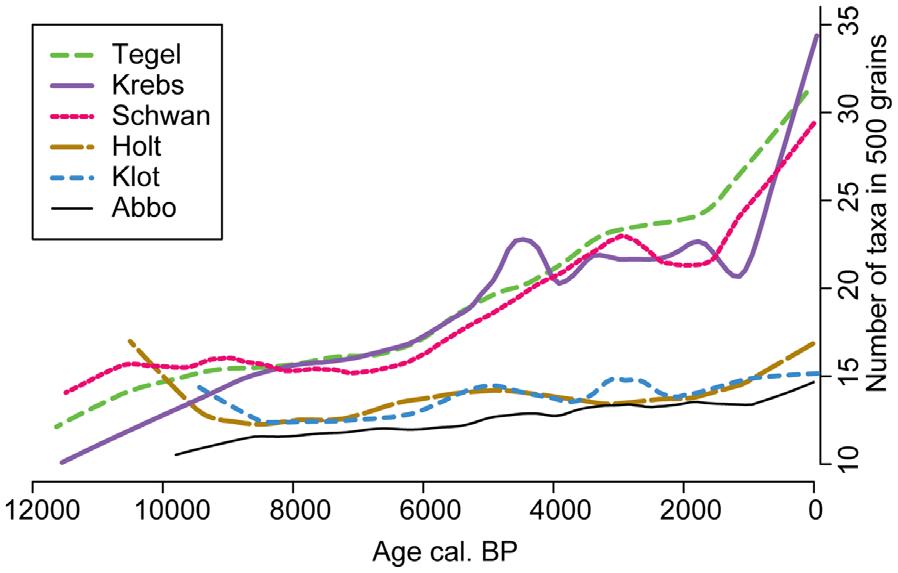
Palynological richness. Number of pollen types encountered per sample based on a count of 500 pollen grains (E(T_500_)) from upland vascular plants (set A), showing only Holocene samples. Lowess smoothers with a span of 0.2 were applied to emphasise trends.

The sample-based palynological richness of upland vascular plants shows different patterns between the two groups of sites, but similar patterns within the groups (Figure 2). The northern sites show little change over the course of the Holocene. Holtjärnen and Klotjärnen start with relatively high values while the northernmost site, Abborrtjärnen, starts with low values and samples from this site maintain slightly lower palynological richness. All southern sites start with a low number of pollen types per samples, which is in some cases even lower than the values obtained for the northern sites. The southern sites increase in palynological richness in tree steps: during the early Holocene, after 6000 cal. BP and with a further increase over the last 1000 years. This pattern does not change when the analysis is run with the dataset excluding pollen types from archaeophytes and neophytes, although the values are somewhat lower for the last 6000 years. The similarity in the pattern of the southern sites shows that all sites portray the same regional development even though site characteristics differ.

**Figure 3:**
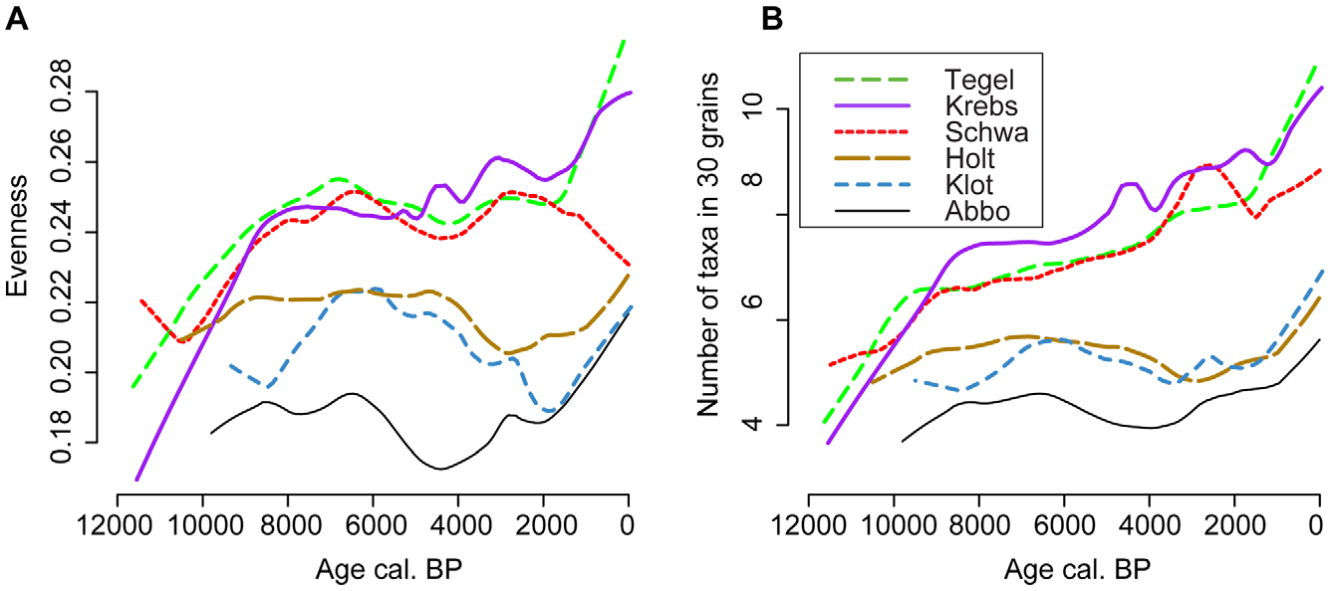
Estimates of pollen sample evenness. A) E_Q_’ indicator based on the slope of the rank abundance plot [36] for taxa exceeding 0.3%; B) Evenness estimated by rarefaction to a count of 30 pollen grains (E(T_30_)). Calculations were based on pollen from upland vascular plants (set A), showing only Holocene samples. A lowess smoother with a span of 0.2 was applied to emphasise trends.

The two different measures of evenness indicate low palynological evenness throughout the Holocene for all sites, with different patterns for the two groups of sites (Figure 3). The overall pattern for the southern sites is similar in both assessments, with lowest evenness for the oldest samples. For the northern sites the detailed patterns differ somewhat between sites. Interesting to note is the 2000 cal. BP drop in the E_Q_’ evenness at Klotjärnen, caused by the reduction of pollen from understory vegetation with the expansion of *Picea abies*.

**Figure 4:**
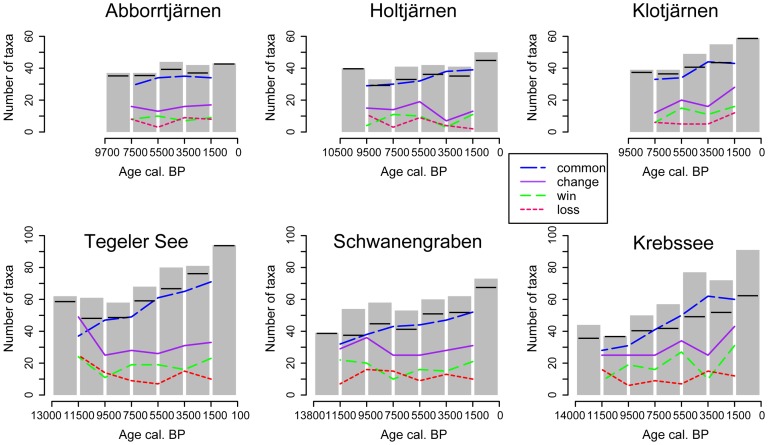
Changes in pollen type diversity between time periods for individual sites. Bars mark the absolute number of taxa in combined samples over time periods of 2000 and 1500 years. Horizontal bars mark the number of taxa based on the rarefaction to the pollen sum of the smallest combined sample per site. Lines indicate the direction of change in taxa composition between periods: common = number of common taxa between two adjacent periods; win = number of taxa gained from one period to the next; loss = number of taxa lost from one period to the next; change = sum of win and loss indicating species turnover. The analysis is based on the pollen types from upland vascular plants (set A).

The overall taxonomic composition changed little through time at the northern sites (Figure 4). The small increase in taxa for the last 1500 years is clearly linked to the opening of the forest and cultivation of crops near Holtjärnen and Klotjärnen. Apart from the high palynological richness in the most recent period, Holtjärnen shows highest richness in the oldest period and Abborrtjärnen for the mid Holocene while Klotjärnen shows a small increase over the last 5000 years.

The southern sites show a general increase in the number of taxa. Over the course of the Holocene the number of pollen types lost from one period to the next is often lower than the number of types gained and in consequence species are accumulating through time (Figure 4). The Tegeler See record shows an almost 50% turnover of species between the Late-Glacial and the early Holocene, while at Schwanengraben there is mainly a gain of taxa at the onset of the Holocene and at Krebssee the number of lost taxa is slightly higher than newly gained taxa. Krebssee shows increased turnover rates towards the most recent time period.

**Figure 5:**
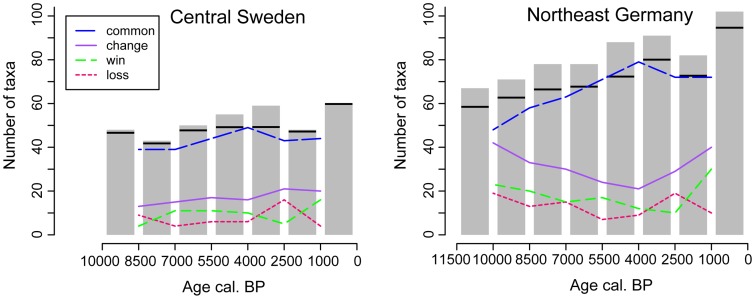
Changes in pollen type diversity in the combined datasets for the northern and southern sites. Bars mark the absolute number of taxa in combined samples over time periods of 1000 and 1500 years. Horizontal bars indicate the number of pollen types in 43363 grains (the smallest number of all bins) allowing comparisons between regions. Lines indicate the direction of change in taxa composition between combined samples: common = number of common taxa between two adjacent combined samples; win = number of taxa gained from one period to the next; loss = number of taxa lost from one period to the next; change = sum of win and loss indicating species turnover. The analysis is based on the pollen types from upland vascular plants without archaeophytes or neophytes (set B).

Combining the three regional sites into one, based on the restricted dataset (set B) reveals additional insights (Figure 5). At the northern sites the number of pollen types is elevated in the oldest bin as well as for the mid Holocene. The expansion of *Picea abies* around 2500 cal. BP appears to be linked to the highest species turnover. Interesting is that this time period shows also the highest loss of pollen types at the combined southern sites. The southern sites also show a peak in shared species around 4000 cal. BP.

**Figure 6:**
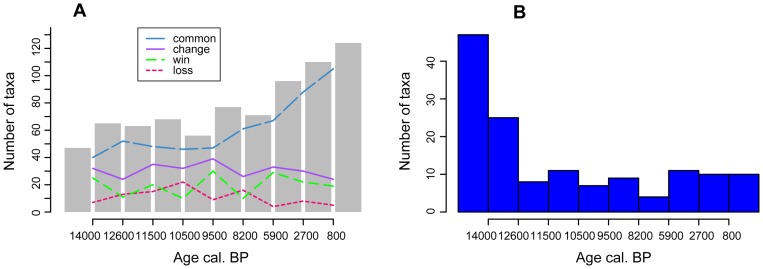
Pollen type diversity and first occurrence in the Berlin/Brandenburg database of selected taxa. A) Bars mark the number of different taxa found per time interval. Lines indicate the direction of change in taxa composition between combined samples: common = number of common taxa between two adjacent combined samples; win = number of taxa gained from one period to the next; loss = number of taxa lost from one period to the next; change = sum of win and loss indicating species turnover. B) Histogram showing the number of taxa that appear for the first time in a given time interval.

The compilation of selected pollen taxa from 113 sites in and around Berlin adds some information on the regional change in pollen taxa diversity through time (Figure 6). The calculation of taxa gained from one period to the next shows four peaks: two smaller at the onset of the Allerød and the Holocene and two higher at 9500 and 5900 cal. years BP. The highest loss of taxa can be seen at 10,500 cal. BP. The regional turnover of typical Late-Glacial pollen taxa to Holocene taxa did not occur at once, but was gradual over the early Holocene. Over the last 6000 years the gain of taxa is higher than the loss resulting in an accumulation of taxa with the highest richness for the most recent period. The histogram indicating the earliest appearance of these pollen types in the region shows that most types were already present in the Late-Glacial (Figure 6b). Higher first occurrences are seen for the earliest Holocene, but also for the periods after 6000 cal. years BP, when mainly pollen from archaeo- and neophytes appear for the first time (see Table S2 in Supporting Information).

**Figure 7:**
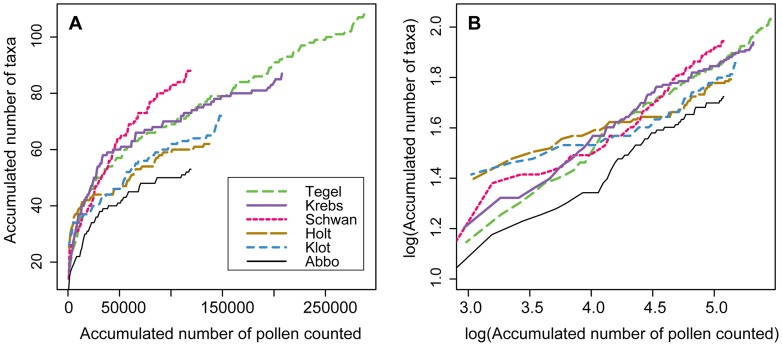
Accumulation of pollen taxa versus accumulated number of pollen counted. The number of pollen taxa accumulated over consecutive samples is plotted against the accumulated number of pollen counted A) in normal space and B) on a log-log transformed scale. Only pollen types from upland vascular plants without archaeophytes or neophytes (set B) were considered.

**Figure 8:**
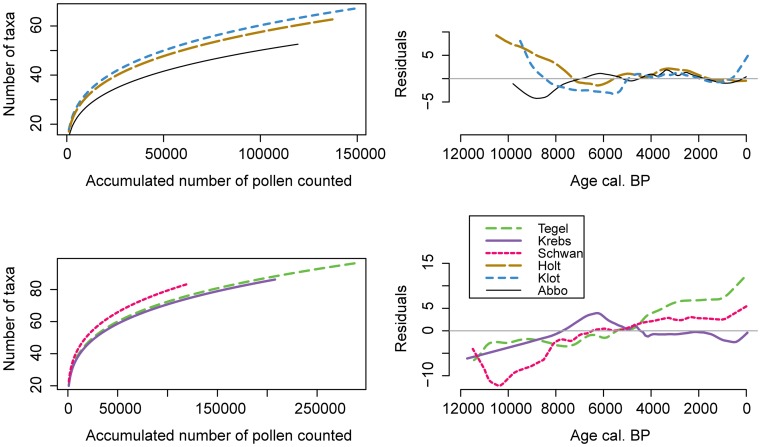
Power functions and residuals of the expected versus observed number of accumulated pollen types. Linear regression models with constant slope and varying intercepts were fitted to the taxa accumulation plot (Figure 7A). Left panels show these power functions in normal space and the right panels show the residuals indicating the difference between the observed and the expected number of accumulated taxa through time. A lowess smoother with a span of 0.1 was applied to emphasise trends. Only pollen types from upland vascular plants without archaeophytes or neophytes (set B) were considered.

The accumulation of taxa versus sampling effort (Figure 7) does not reach an asymptote at any of the sites, but can be described by a power function. Although pollen types from archaeo- and neophytes are excluded, samples dating to the last few hundred years show an increase in accumulated taxa. The log-transformed accumulation curves show linear relationships with an overall slope of *w* = 0.27 and intercepts between 0.35 and 0.55. The individual curves often show deviations from the overall rate of increases or can be divided into sections with different increase as can be seen for Holtjärnen, Klotjärnen and Schwanengraben. However, also these diagrams follow the overall rate of taxa accumulation for the major part of the record. It appears that the rate of taxa accumulation is mainly determined by the pollen count, influenced by pollen sample evenness [40] and potentially the true diversity of pollen types in the samples. Thus, by subtracting the overall relationship between taxa accumulation and pollen count, the deviation from that trend should mainly reflect changes in evenness and palynological diversity. As evenness can be estimated independently this allows an evaluation of changes in pollen type and potentially floristic diversity around the site through time (Figure 8).

At the northern sites, the power functions describe the increase in pollen taxa accumulation extremely well for the last 6000 years, but underestimate the number of taxa in the earliest samples from Holtjärnen and Klotjärnen. Reversely the three southern sites and also Abborrtjärnen contain fewer than expected taxa in the oldest samples after the onset of the Holocene.

## Discussion

### Did Immigration Influence Diversity?

All measures used here show that pollen type richness is lowest at the northern sites and highest at the southern sites. This agrees with the general impoverishment of the vascular plant flora along this latitudinal and temperature gradient and shows that palynological richness captures interregional differences in floristic diversity. The pollen diagrams from the three northern sites show that there is no substantial increase in palynological richness through time except for the last 1000 years, which is due to human land-use. The southern sites show changes in palynological richness and in the rate of taxa accumulation, which in part are due to agricultural practice that started in this region around 6000 cal. BP [51] and influenced the regional species pool as well as the landscape structure. The lack of these features at the northern sites shows that here the regional species pool did not expand through time. Hence there is no evidence to indicate that slow plant migration had a strong influence on the floristic diversity at these northern sites on time scales of hundreds to thousands of years. This does not mean that there may not be any delay in the arrival of individual species due to dispersal biology and slow population growth in the order of hundreds of years. Birks and Birks [52], for example, show a 450 years delay for the arrival of *Betula pubescens* in western Norway after the onset of the Holocene warming. Here we did not evaluate individual species but the whole assemblage based on presence and absence of pollen types, without making interpretations on pollen coming from local sources versus long distance transported pollen [53]. Due to the high pollen production of most European trees their pollen is often found already in the early Holocene. Thus, their respective pollen taxon entered the taxa accumulation curve early, regardless of interpretations on the timing of their arrival. Therefore, the taxa accumulation curve is mainly determined by the appearance of herbaceous pollen.

Pollen diagrams depicting the successive arrival of trees led early palaeoecologists to suspect species would be lagging behind the spatial expansion of their climate envelop at the beginning of the Holocene [54]. Based on the comparison of the distribution of climate parameters with the distribution of species, Svenning and Skov [15] argue that on average European trees realise only 40% of their potential range, providing new support for the existence of migrational lag [16]. While supporting these findings, Normand et al. [17] find little evidence that the distribution of plants in northern Europe could be explained by a slow spread out of presumed LGM distributions. Thus the distribution of plants in previously glaciated areas of northern Europe probably established quickly after the onset of the Holocene and following deglaciation, with little discernible effect of a migrational lag. Southern Europe, on the other hand, holds many plants that could thrive further north [17] and some of them have been introduced beyond their natural ranges where they are naturalised. The question remains why these species have not managed to reach other areas. If the flora in northern Europe has changed little over the course of the Holocene, its composition may be dominated by those that managed to arrive early. On the other hand, little is known about the Holocene distributional changes of the southern deciduous oaks like *Quercus frainetto* as they share the same pollen type with species that are wider distributed. Thus it is difficult to infer if these species survived the LGM in particular locations and later expanded their distribution over large areas as we can reconstruct it for northern Europe. Alternatively these populations could have merely expanded out of scattered groups of trees that occurred during the LGM in approximately the same area as today. Such knowledge could inform on the question on whether these species would spread and eventually fill their potential ranges if given enough time. It may be that many southern species did not lag in their migration, but did not spread from their LGM distributions.

### Early Holocene Palynological Richness

At the three northern sites land became only available for the colonization of plants during the early Holocene, which occurred in a different manner at the different sites. The earliest samples of the three northern sites contain only a few pollen types that disappear entirely from the overlying samples. Overall the pioneer vegetation at the northern sites was mainly composed of the same plants that are found in the boreal forest around the sites today. At the southern sites, the onset of sedimentation started at different times after deglaciation and the early pioneer phases occurred in conjunction with Late-Glacial climate oscillations making it difficult to separate the role of climate on species diversity and palynological richness. The species composition changed strongly at the onset of the Holocene with several species disappearing and many becoming rare for at least a few thousand years (Figure 4, Table S2 in Supporting Information).

The oldest samples from Holtjärnen and Klotjärnen show high palynological richness and a higher than expected number of taxa (Figure 8). This may be caused by a large proportion of open vegetation near the sites during the early Holocene. Decreasing trends in palynological richness over the early Holocene are found in diagrams from western Denmark and southern Sweden [27], [55]. Seppä [56] showed how the expansion of pine woodland around a site in northern Finland lowered palynological richness. Around Abborrtjärnen pine forest quickly established and palynological richness in the oldest samples is low. Woodland had already established by the time Klotjärnen emerged from the Baltic Sea, but the shore remained close to the lake for some time and through isostatic uplift new land became gradually available for plants to colonize. The pollen diagram from Holtjärnen is documenting the arrival of plants from a long distance away and while populations were building up, the landscape remained partly open. Interesting to note is also that the oldest samples from Holtjärnen contain more pollen from *Ulmus* and *Corylus avellana* than the youngest samples from the site, indicating that these thermophilous elements were already part of the early-Holocene vegetation mosaic dominated by *Betula* and later by *Pinus sylvestris*. Thus, in particular these two species with different dispersal mechanisms show no delay in their arrival.

The opposite process determined palynological richness at the southern sites. Here *Betula* species and *P. sylvestris* formed woodlands during the Allerød period. Many of these woodlands reduced in size, but survived in sheltered places during the cold Younger Dryas period [57]. With the onset of the Holocene warming these populations could quickly expand to dominate the landscape together with a few other Late-Glacial survivors. Long distance dispersed propagules found new ground near Holtjärnen and Klotjärnen with little competition for populations to expand, in the south new arrivals had to compete with the established plants for resources. While this probably did not prevent the establishment of a species, it may have slowed population expansion.

### Holocene Change in floristic and Landscape Diversity

When introducing the rarefaction technique to pollen analysis Birks and Line [29] state that palynological richness might be influenced by physical features of the site. It is thus surprising to see the that E(T_500_) curves for the southern sites run parallel for most of the Holocene even though the characteristics of these sites are very different, ranging from a forest hollow to the embayment of a large lake. Also palynological richness estimated for pollen diagrams from three Estonian peat-lands ranging from 30 to over 200 ha show no major effect of basin size [58].

The taxa accumulation curves, on the other hand, reflect local site specific differences more strongly. Here the smallest site yields the largest number of pollen types per count and its change from a shallow lake to bog had a large effect on the accumulation of new taxa. Assuming that the linear relationship of the taxa accumulation curve in log-log space is largely a sampling effect, the residuals can inform on changes in evenness and species immigration. After accounting for pollen types coming from archaeophytes and neophytes the taxa accumulation curves for Krebssee and Schwanengraben follow well the predicted values for the last 8000 years, while their E(T_500_) curves increase markedly around 5000 cal. years BP. This rise in palynological richness is not caused by the early appearance of archaeophytes, but by proportional changes in previously present taxa. Thus, sample based palynological richness seems to be a good indicator of changes in landscape diversity but holds little information on the size of the regional species pool. At Tegeler See the residuals of the observed versus the predicted taxa accumulation rise after 5000 cal. years BP, while the curves for the other two southern sites remain flat. This pattern for Tegeler See may be site-specific, possibly connected to the regional increase in new man-made environments.

An increasing number of pollen types shared between consecutive time periods (Figures 4, 5) can be observed for Tegeler See and Krebssee. This is also visible in the selected taxa dataset from Berlin/Brandenburg sites (Figure 6). These patterns are partly caused by the addition of pollen taxa from cultivated species and associated weeds, but also represent the more consistent occurrence of pollen types that were previously encountered. The latter is often also connected to the opening of the forest for agriculture, increasing the habitat for many herb taxa (apophytes) that were abundant during the Late-Glacial, but rare during the early to mid-Holocene dominance of trees. However, the landscape diversity may also have slowly increased due to autogenic processes. On the nutrient poor sandy soils, the pine dominated forest that developed quickly at the onset of the Holocene may have possessed a large resilience towards the expansion of thermophilous trees. Over time fire and wind throw created gaps that could be sized by previously rare species and thus for example *Quercus* could slowly increase in abundance changing in turn the environment for other plants [59].

Also temperature changes through the Holocene may have influenced floristic diversity at the investigated sites, which may be reflected by the mid-Holocene maximum number of pollen types observed for the northern sites (Figure 5). In this respect the loss of taxa around 2500 cal. BP could also be interpreted as a reaction to the late Holocene climate cooling, which is difficult to separate from the effect that the expansion of *Picea abies* had on the boreal ecosystem [60]. A climatic cause would explain the parallel reduction of pollen types in the diagrams from Brandenburg during the first expansion of *Fagus sylvatica*. However, here this hypothesis has to compete with the consideration that this effect may have been caused by a reduced settlement activity at the transition from the Bronze to the Iron Age.

### Species Accumulation Curves

The accumulation of pollen taxa is compared against accumulated pollen counts, which is a measure of sampling effort. However, each sample represents a snapshot in time. Plants that were distant to the sampling site or rare in its surrounding at one time may be closer to it or abundant during a consecutive time period and release more pollen to the site. Thus, in effect the pollen taxa versus pollen count relationship constructed here is effectively a species-time relationship. Preston [61] suggested that species-time relationships would work similarly to species area curves assuming a linear relationship in log-log space [48]. Recently species time curves have received attention on ecological [62], [63] and geological time scales [48], [64]. However, they have so far not been explored for pollen data on the late Quaternary timescale, which lies in between long ecological observations of several decades and geological time measured in thousands to millions of years. The particular problems of pollen data with regards to undefined space and the influence of evenness are setting these datasets apart from long term ecological datasets. On the other hand, the lack of speciation and the rarity of extinction differentiate late Quaternary data from longer geological time series.

The exponent *w* = 0.27 for the power function found here is well in the range of the literature for species-time relationships from long ecological datasets [63]. As the number of pollen types per pollen count depends on the pollen sample evenness this should influence the exponent *w*, being smaller at higher evenness and larger at lower evenness. This is exemplified by the positive residuals for Holtjärnen and Klotjärnen, coinciding with higher evenness, while the negative residuals for the southern sites are characterized by lower evenness (Figures 3 and 8). The evenness measures used here are biased towards taxa with high abundance and the trends in taxa accumulation in log-transformation may yield further insight in evenness changes. An even vegetation composition will mean that many taxa will be found in one sample and only few more in the consecutive sample. In an uneven vegetation with the same number of species, chances are high that different taxa are captured in consecutive samples resulting in a steeper slope *w*. This effect of evenness on the slope in species-time relationships has so far received little attention. However, the negative relationship between mean annual richness and slope *w* in species-time relationships from ecological datasets compared by White et al. [63] indicates that this effect is of general importance.

### Conclusions

At three sites in central Sweden palynological richness has not increased over the course of the Holocene and species composition has changed only little. Thus there is no evidence for increased taxonomic diversity due to delayed immigration of species. At the southern sites stepwise increases in palynological richness are observed for the early Holocene around 6000 and 1000 years ago. Here humans have actively introduced agricultural and other useful plants with associated weeds starting around 6000 years ago. In two out of three sites pollen types from these archaeophytes and neophytes can account for the increase in newly appearing taxa.

The Late-Glacial vegetation development at the southern sites led to an early-Holocene advantage for boreal taxa, which at least reduced the rate of population expansion for newcomers and lead to a low vegetation evenness and possibly diversity. Taxa accumulation curves from pollen diagrams can help evaluating past changes in vegetation structure and diversity.

## Supporting Information

Figure S1 **Percentage pollen diagrams showing a standardized set of selected taxa.** The dotted lines in the diagrams from the southern sites mark the Holocene/Late-Glacial boundary as determined from pollen stratigraphy, which corresponds to slightly different ages at the three sites due to uncertainties in the age models.
(PDF)

Table S1 **List of pollen types associated with archaeophytes and neophytes and therefore removed from dataset A, crating the restricted dataset B.**
(PDF)

Table S2 **Occurrences of selected pollen and spore taxa from 113 sites in Berlin/Brandenburg.** Approximate ages of Firbas zones boundaries for Berlin/Brandenburg in years cal. BP: 1/2 = 14000; 2/3 = 12600; 3/4 = 11500; 4/5 = 10500; 5/6 = 9500; 6/7 = 8200; 7/8 = 5900; 8/9 = 2700; 9/10 = 800.
(PDF)
