# Supplementary material for: Exploring Holocene Changes in Palynological Richness in Northern Europe – Did Postglacial Immigration Matter?
Source: PLoS One. 2012 Dec 11;7(12):e51624. doi: 10.1371/journal.pone.0051624 (PMC3519870; doi:10.1371/journal.pone.0051624)
Supplement: Figure S1 — Percentage pollen diagrams showing a standardized set of selected taxa. The dotted lines in the diagrams from the southern sites mark the Holocene/Late-Glacial boundary as determined from pollen stratigraphy, which corresponds to slightly different ages at the three sites due to uncertainties in the age models. (PDF) [file pone.0051624.s001.pdf]

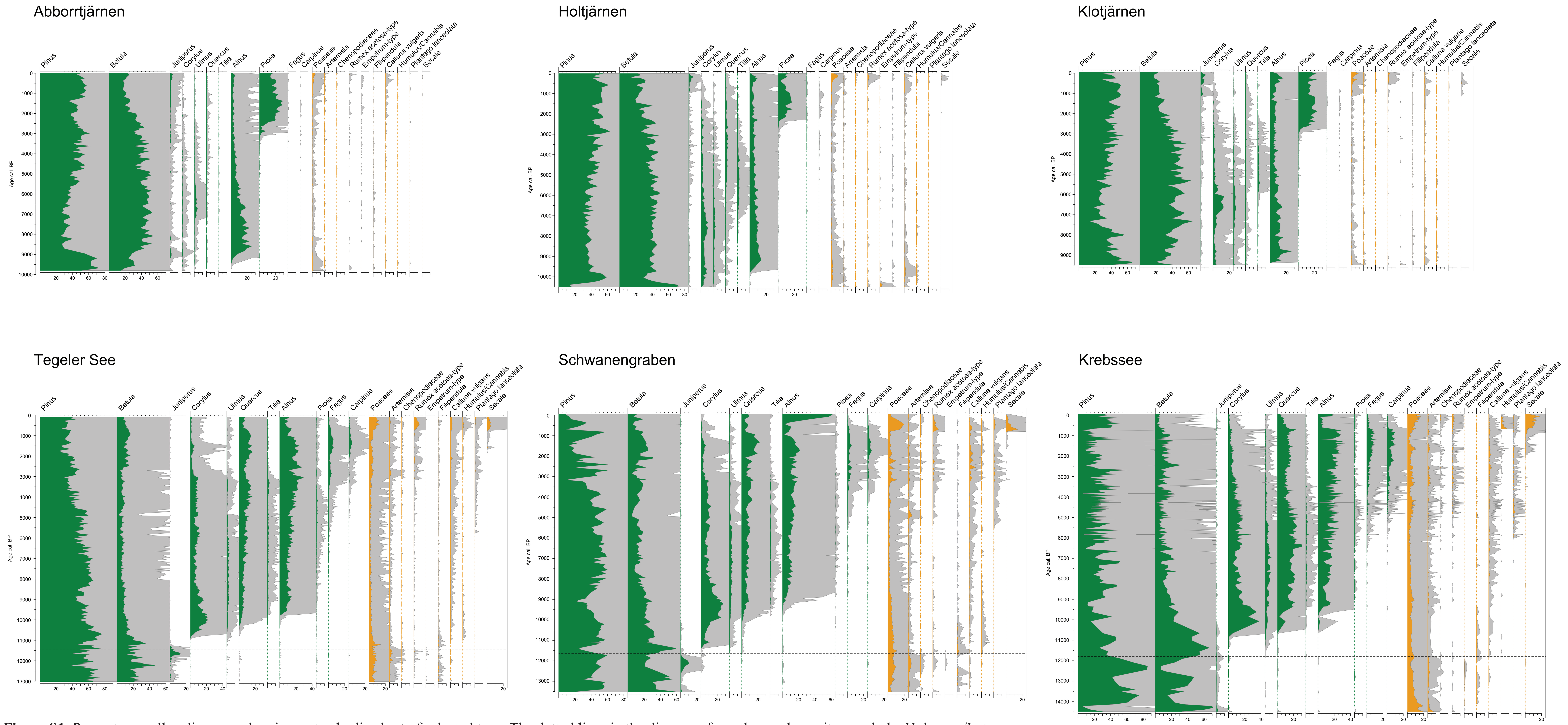

Figure S1. Percentage pollen diagrams showing a standardized set of selected taxa. The dotted lines in the diagrams from the southern sites mark the Holocene/Late-Glacial boundary as determined from pollen stratigraphy, which corresponds to slightly different ages at the three sites due to uncertainties in the age models.
