# Supplementary material for: Exploring Holocene Changes in Palynological Richness in Northern Europe – Did Postglacial Immigration Matter?
Source: PLoS One. 2012 Dec 11;7(12):e51624. doi: 10.1371/journal.pone.0051624 (PMC3519870; doi:10.1371/journal.pone.0051624)
Supplement: Table S1 — List of pollen types associated with archaeophytes and neophytes and therefore removed from dataset A, crating the restricted dataset B. (PDF) [file pone.0051624.s002.pdf]

**Table S1.** List of pollen types associated with archaeophytes and neophytes and therefore removed from dataset A, crating the restricted dataset B.

*Aesculus*
*Aphanes*
*Avena*-type
*Castanea*
*Centaurea cyanus*
*Cerealia* undiff.
*Cerealia*-type
*Delphinium*
*Echium*
*Fagopyrum*
*Fallopia*
*Hordeum*-type
*Impatiens*
*Jasminum*
*Linum usitatissimum*
Malvaceae
*Nigella*
*Papaver argemone*
*Papaver rhoeas*-type
*Platanus*
*Scleranthus annuus*
*Secale*
*Triticum*
*Verbena*
*Zea mays*
