# Supplementary material for: Exploring Holocene Changes in Palynological Richness in Northern Europe – Did Postglacial Immigration Matter?
Source: PLoS One. 2012 Dec 11;7(12):e51624. doi: 10.1371/journal.pone.0051624 (PMC3519870; doi:10.1371/journal.pone.0051624)
Supplement: Table S2 — Occurrences of selected pollen and spore taxa from 113 sites in Berlin/Brandenburg. Approximate ages of Firbas zones boundaries for Berlin/Brandenburg in years cal. BP: 1/2 = 14000; 2/3 = 12600; 3/4 = 11500; 4/5 = 10500; 5/6 = 9500; 6/7 = 8200; 7/8 = 5900; 8/9 = 2700; 9/10 = 800. (PDF) [file pone.0051624.s003.pdf]

Table S2. Occurrences of selected pollen and spore taxa from 113 sites in Berlin/Brandenburg.

Approximate ages of Firbas zones boundaries for Berlin/Brandenburg in years cal. BP:
1/2=14000; 2/3=12600; 3/4=11500; 4/5=10500; 5/6=9500; 6/7=8200; 7/8=5900; 8/9=2700; 9/10=800.

Pollen / spore taxon	Firbas zone									
	1	2	3	4	5	6	7	8	9	10
Aesculus										1
Bidens-type										1
Buxus										1
Erodium										1
Herniaria-type										1
Lycopsis										1
Osmunda										1
Papaver rhoeas-type										1
Platanus										1
Rubus-type										1
Verbena										1
Castanea									1	1
Fagopyrum									1	1
Impatiens									1	1
Linum usitatissimum									1	1
Malvaceae									1	1
Mercurialis annua									1	1
Sagina									1	1
Scleranthus annuus									1	1
Viola arvensis									1	1
Convolvulus arvensis								1	1	1
Hydrocharis									1	
Hydrocotyle								1	1	1
Juglans								1	1	1
Ledum								1	1	1
Radiola								1		1
Stratiotes								1	1	1
Symphytum								1	1	1
Linum							1		1	1
Euphorbia							1	1	1	1
Gentiana pneumonanthe-type								1	1	
Iris							1	1	1	1
Lonicera								1	1	
Sambucus							1	1	1	1
Thesium								1	1	
Drosera intermedia						1			1	1
Drosera rot./angl.						1		1	1	1
Sagittaria						1		1	1	1
Scleranthus perennis						1		1	1	1
Vitis						1		1	1	1
Andromeda					1				1	1
Anthoceros laevis-type					1			1	1	1
Cuscuta europaea-type					1				1	1
Epipactis palustris-type					1				1	1
Mercurialis perennis						1	1	1	1	1
Rubus chamaemorus								1		
Dipsacaceae						1	1	1		1
Riccia				1				1	1	1
Cornus sanguinea					1	1	1	1	1	1
Hedera					1	1	1	1	1	1
Pedicularis palustris-type			1					1	1	1
Taxus					1	1	1	1	1	1
Spergularia-type				1		1		1	1	1
Acer				1		1	1	1	1	1
Anthericum				1		1	1	1	1	1
Echium			1						1	1
Scabiosa				1		1	1	1	1	1
Alisma plantago-aquatica-type			1			1	1	1	1	1
Calystegia				1	1	1	1	1	1	1
Centaurea cyanus	1							1	1	1
Frangula				1	1	1	1	1	1	1
Knautia		1					1		1	1
Lycopodium annotinum-type		1				1	1	1	1	1
Papaver argemone		1							1	1
Polygala						1		1		
Rhamnus cathartica				1	1	1	1	1	1	1
Solanum dulcamara				1	1	1	1	1	1	1
Viburnum				1	1	1	1	1	1	1
Viscum				1	1	1	1	1	1	1

Pollen / spore taxon	Firbas zone									
	1	2	3	4	5	6	7	8	9	10
Lycopodium clavatum-type			1		1	1	1	1	1	1
Anthoceros punctatus-type	1					1		1	1	1
Sedum		1		1		1	1	1	1	1
Cladium			1	1	1	1	1	1	1	1
Lycopodium inundatum		1		1		1		1	1	1
Lysimachia			1	1	1	1	1	1	1	1
Scheuchzeria		1		1		1		1	1	1
Succisa			1	1	1	1	1	1	1	1
Teucrium-type			1							1
Campanula		1	1					1	1	1
Jasione		1		1	1	1	1	1	1	1
Lythrum		1		1	1	1	1	1	1	1
Thelypteris palustris		1		1	1	1	1	1	1	1
Utricularia		1		1	1	1	1	1	1	1
Polygonum convolvulus-type	1		1			1	1	1	1	1
Polygonum persicaria-type	1			1	1	1		1	1	1
Polypodium		1	1	1		1	1	1	1	1
Valeriana		1	1	1		1	1	1	1	1
Xanthium	1		1		1	1	1	1	1	1
Campanulaceae		1	1	1	1	1	1	1	1	1
Rumex subg. Rumex		1	1	1	1	1	1	1	1	1
Lemnaceae	1	1			1	1	1	1	1	1
Nuphar		1	1	1	1	1	1	1	1	1
Polygonum amphibium		1	1	1	1		1	1	1	1
Polygonum aviculare-type		1	1	1	1	1	1	1	1	1
Pteridium		1	1	1	1	1	1	1	1	1
Plantago maritima-type	1	1	1			1	1	1	1	1
Ephedra distachya-type		1	1	1		1	1	1		1
Gentianaceae	1	1	1			1		1	1	1
Centaurea jacea-type	1	1	1	1		1	1	1	1	1
Epilobium	1	1	1	1		1	1	1	1	1
Plantago lanceolata	1	1	1	1		1	1	1	1	1
Armeria	1	1							1	1
Botrychium	1	1	1	1	1	1	1	1	1	1
Chenopodiaceae	1	1	1	1	1	1	1	1	1	1
Cirsium-type	1	1	1	1	1	1	1	1	1	1
Brassicaceae	1	1	1	1	1	1	1	1	1	1
Hippophae	1	1	1	1	1	1	1	1	1	1
Juniperus	1	1	1	1	1	1	1	1	1	1
Lotus	1	1	1	1	1	1	1	1	1	1
Lycopodium selago	1									1
Menyanthes	1	1	1	1	1	1	1	1	1	1
Nymphaea	1	1	1	1	1	1	1	1	1	1
Ophioglossum	1	1	1	1	1	1	1	1	1	1
Plantago major/media	1	1	1	1	1	1	1	1	1	1
Rumex acetosa-type	1	1	1	1	1	1	1	1	1	1
Sanguisorba officinalis	1	1	1	1	1	1	1	1	1	1
Sparganium erectum-type	1	1	1	1	1	1	1	1	1	1
Thalictrum	1	1	1	1	1	1	1	1	1	1
Typha angustifolia	1	1	1	1	1	1	1	1	1	1
Typha latifolia-type	1	1	1	1	1	1	1	1	1	1
Urticaceae	1	1	1	1	1	1	1	1	1	1
Centaurea scabiosa-type	1	1	1	1	1		1	1	1	1
Helianthemum	1	1	1	1	1		1	1	1	1
Parnassia	1	1	1	1	1		1	1	1	1
Ephedra fragilis-type		1	1	1				1		1
Myriophyllum spic./vert.	1	1	1	1	1	1		1	1	1
Gypsophila	1	1	1	1	1	1	1	1		1
Polygonum bistorta-type	1	1	1	1	1	1			1	1
Sanguisorba minor	1	1	1	1					1	1
Geum-type		1	1	1					1	
Arctostaphylos	1	1	1	1		1	1	1		
Larix	1	1	1							1
Selaginella selaginoides	1	1	1	1		1	1			
Dryas-type		1	1	1	1					
Empetrum	1	1	1	1	1	1				
Saxifraga hirculus-type	1	1	1				1			
Hippuris	1	1	1	1		1				
Gymnocarpium		1		1						
Myriophyllum alterniflorum		1	1	1						
Polemonium	1	1	1							
Saxifraga	1		1							
Saxifraga aizoides-type	1	1	1							
